# Resolving anxiety-like behaviour inconsistencies in the elevated plus maze by tracking exploration depth and timing

**DOI:** 10.3758/s13428-025-02738-8

**Published:** 2025-06-30

**Authors:** Matthew D. Zelko, Stephen R. Robinson, Elisa L. Hill-Yardin, Helen Nasser

**Affiliations:** 1 School of Health and Biomedical Sciences, RMIT University, Bundoora, VIC 3083 Australia; 2 School of Psychology and Public Health, La Trobe University, Bundoora, VIC 3083 Australia; 3 Epigenes Australia Pty Ltd, Mooroolbark, VIC 3138 Australia; 4 Institute for Breathing and Sleep (IBAS), Austin Health, Heidelberg, Victoria 3084 Australia; 5 Department of Anatomy & Physiology, The University of Melbourne, Parkville, VIC 3010 Australia

**Keywords:** Anxiety-like behaviour, Elevated plus maze, Exploration, Avoidance, Novel exploration growth

## Abstract

The anxiety-like behaviour provoked by the elevated plus maze is primarily measured by the degree to which a rodent explores or avoids the risker, unenclosed arms. Measures such as arm entries and total time spent within the arm are conventionally used, but their analysis often produces inconsistent inferences about the level of anxiety-like behaviour being observed. This inconsistency occurs because the measures do not correlate with one another, raising the question of how validly they capture both the exploratory and avoidance motivations that typify anxiety-like behaviour. Given this inconsistency, we propose a new measure, Novel Exploration Growth, that captures avoidance and exploratory behaviours within the maze. The growth of novel exploration tracks the first visit to discrete areas of the maze over time. The absolute amount of novel exploration, combined with the phasic nature of the exploration, reveals behavioural phenotypes with both avoidance and exploration quantified in a single time series. By addressing both motivations evoked by the maze through a single coherent measure, we provide a superior estimation of anxiety-like behaviour and resolve inconsistencies that arise when applying conventional measures alone.

**Supplementary Information:**

The online version contains supplementary material available at 10.3758/s13428-025-02738-8.

## Introduction

The elevated plus maze (EPM) is a popular paradigm for testing rodents’ anxiety-like behaviour (ALB). The EPM consists of four elevated arms, two of which are surrounded by high walls, known as closed arms. The remaining two arms, known as the open arms, are not enclosed. These two distinct arm types allow researchers to observe whether the rodent seeks safety in the closed arms or risks detection by exploring the open arms. (Carola et al., 2002; Leo & Pamplona, 2014). Avoidance of open arms in favour of the safety of closed arms is considered to indicate a greater degree of ALB. In contrast, exploration of the open arms indicates a lower degree of ALB. Exposure to the maze, therefore, evokes a conflict between the innate motivations of safety and exploration, characterising the ALB as an indicator of emotional state (Hoffman et al., 2022; Perusini & Fanselow, 2015).

Several measures have been used to compare differences in preferencing of an arm type. The standard measures include the total number of entries and time spent in an arm type. As these measures lack temporal sensitivity, researchers also track the initial latency to enter an arm type (Walf & Frye, 2007). However, using latency to enter an arm type assumes that every entrance into an arm is comparable, ignoring differences in the depth of exploration that follows it. This limitation can result in an early, shallow entry into the open arms, being interpreted as lower anxiety-like behaviour than a delayed but deeper exploratory entry (Tejada et al., 2010).

Alongside the lack of temporal or spatial sensitivity of these conventional measures, it is unclear how to prioritise outcomes from these measures when making inferences about the level of ALB being observed in rodents. For example, a rodent may only enter the open arms a few times but may spend more time investigating the environment in depth. Ethologically, this rodent displays high amounts of exploratory drive. Conversely, another rodent may make many shallow entries into the open arms but never fully commit to exploring them beyond the entry boundary, a pattern that should be inferred as a higher degree of ALB. Despite this, the greater number of entries into the open arms would typically be interpreted as a lower degree of ALB compared to the former exploratory counterpart. Thus, the most appropriate selection of available behavioural measures is contingent upon potentially subjective decisions determining which behaviour best typifies ALB. This reliance on subjectivity can lead to difficulty in replicating outcomes or translating intervention effects to human populations.

The present paper overcomes the abovementioned limitations by providing a single, coherent measure of rodent exploratory and avoidant behaviours in the EPM. Using a new measure of ALB, novel exploration growth (NEG), safety and exploration motivational preferences are observed and differentiated into qualitatively distinct behavioural phenotypes. By measuring NEG over time, each behavioural phenotype is mapped to an appropriate level of ALB, allowing justified and replicable inferences to be made about the traversal behaviours of rodents in the EPM.

ALB levels are inferred from the NEG using a multistage analysis workflow. This workflow includes collecting and preprocessing tracking data, which is then analysed using Bayesian change-point regression and Bayesian generalised additive model (GAM) regression. Change-point analysis is used to identify the phases of exploratory behaviour during testing. These phases are then analysed using Bayesian GAM regression to estimate the effects of interest (e.g., between phenotypes or arm types).

To demonstrate how to apply the proposed workflow, distinct behavioural phenotypes were simulated across the entire EPM and for each arm type and their NEGs were analysed. In addition, we compared the phenotypes across the conventional measures to compare the difference in ALB level inferred from these measures and NEG. This comparison highlights the incongruence between these conventional measures and demonstrates how NEG, as a unified and coherent measure, remedies these inconsistencies.

### Novel exploration growth as a unified measure of exploration and avoidance

NEG is measured by tracking the first physical visit to discrete areas of the EPM. The arena is divided into a unidimensional grid of 1-cm rectangles similar to those used in previous studies (Costa & Tinós, 2016; Salum et al., 2000). The initial visit to each 1-cm-deep rectangle is recorded, and unique visits are aggregated over time and expressed as a percentage of the total grid units of the arm type of interest or the entire maze. For example, if a rodent has physically visited all the available grid rectangles in the closed arms, then closed arm NEG equals 100% as it has thoroughly explored both arms. Conversely, if the rodent has only visited half of the available rectangles in the open arms at the same point in time, their open arm NEG equals 50%. Critically, since total NEG is a composite of the two arm types, this rodent has explored 100% of half the maze and 50% of the other half, meaning it has explored 75% of the total maze.

The choice to use the first visit to each location is based on the utility of the existing latency to enter the arm measure to the entire arm or maze. This utility exists because there is a need to distinguish between naïve exploration and informed preference when evaluating the motivation to enter an arm type. In this dichotomy, the first visit is considered naïve, whereas subsequent visits are informed. This distinction links the ALBs observed in the EPM test to foraging behaviours in wild-type rodents. Foraging behaviour encompasses any action taken to locate and exploit a food source (Davidson et al., 2019; Owen-Smith et al., 2010). Foraging behaviour, particularly in new environments, powerfully evokes the conflict between safety and exploration that typifies rodent ALB in the EPM (Lima & Dill, 1990; McArthur et al., 2014). Theoretically, foraging decisions are modelled using the Optimal Foraging Theory, which explains movement as the result of an attempt to maximise resource accumulation while minimising resource expenditure (Pyke, 2019; Stephens, 2008).

A key component of Optimal Foraging Theory is the Marginal Value Theorem, which predicts movement between resource patches (Charnov, 1976; King & Marshall, 2022; Pyke & Starr, 2020). Briefly, this theory predicts how long an animal should remain in a patch with resources when deciding whether to stay or move to novel patches that may offer greater resources. For example, when that resource is food, optimal foraging occurs when net energy expenditure is minimised while also maximising energy gains. This optimisation can only be achieved when the animal correctly predicts the resource value of the current patch and any possible patches to which it may travel. This decision inherently includes a degree of uncertainty that is proportional to the novelty of the possible destinations. Critically, this uncertainty usually incorporates the risk of predation when assessing movement patterns (Found, 2022; Hintz & Lonzarich, 2018; Křivan, 1996).

Rodents signal a greater predicted predation risk by shifting from cautious exploration to behaviours such as surveillance, avoidance, freezing and escape. Theoretically, ALBs occur in the absence of a predator but are typified by the motivation to minimise the likelihood of an encounter (Hoffman et al., 2022; Mobbs et al., 2018; Rodgers et al., 1997). Given this definition, ALBs are considered a cost to net energy gain, resulting in suboptimal foraging. Thus, if the net energy of a foraging activity is a function of energy expenditure and energy gained, both are modified due to slower accumulation and increased non-foraging behaviours such as surveillance and caution.

The utility of NEG as a measure of ALB relies on the assumption that a rodent within the EPM is acting as an optimal forager in a novel environment. When a rodent anticipates a high threat risk, it will avoid exploring the open arms, as this region poses the highest risk of encountering a predator. When the expected risk of predation is low, rodents freely explore the available environment to evaluate possible foraging opportunities. Critically, the expected energy gain from the open arms is nonzero when the arms are novel to a naïve rodent; however, this value diminishes once rodents are informed of the lack of foraging opportunities through exploration. Consequently, exploration reduces the relative expected net energy gain of open-arm areas compared to the closed arms. This deficit exists because, while both arm types lack foraging opportunities, the closed arms provide greater proportional safety than the open arms, so fewer ALBs must be performed in them, reducing energy expenditure. This transition from naïve to informed, and its impact on the value of the arm types, means that an emphasis should be placed on the first visit to a novel location.

**Fig. 1:**
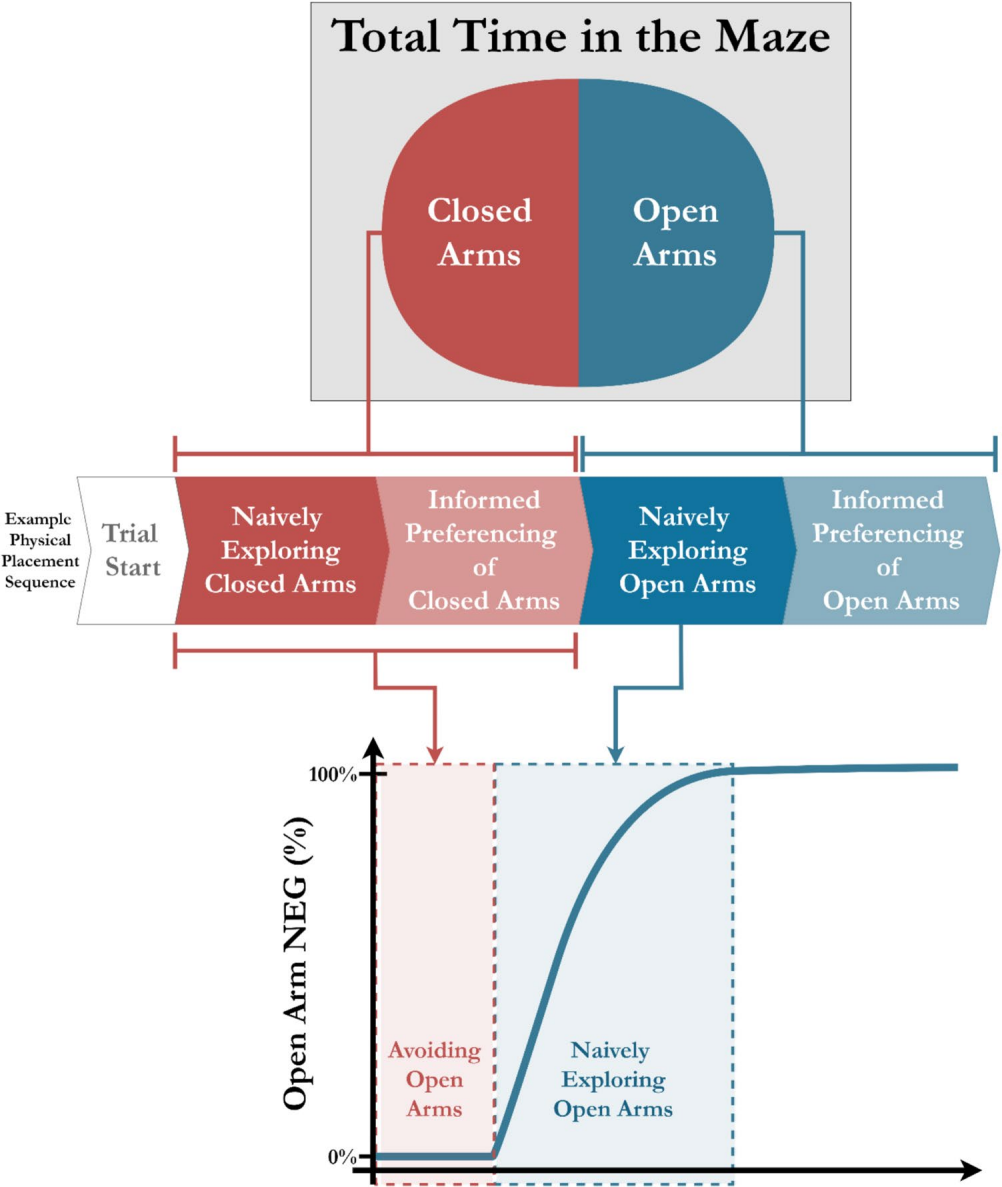
Mapping of time spent in the open arms of the EPM to novel exploration growth using an example testing sequence. The total time in the maze is primarily split between being physically placed within either the open or closed arms. Using an example sequence of physical placement, we show that when a rodent is naively exploring or informedly preferencing the closed arms, they are avoiding the open arms. Similarly, time spent in the open arms is also split into two states: naïve exploration (first visits to discrete areas) and informed preferencing (subsequent visits). As shown in the bottom panel, novel exploration growth (NEG) curves capture both avoidance (shown by flat regions) and exploration (shown by growth regions) of, in this example, the open arms, as a unified indicator of the approach-avoidance conflict prompted by testing in the EPM

The transition from naïve to informed creates a dichotomy within the time spent in the open arms. Conventionally, time spent in the open arms is considered to have the same value across the testing period and to be generated by a single motivation. In contrast, we propose that time spent in the open arms is nonlinear across time and is generated by two motivations: novel exploration and informed preferencing (see Fig. 1). Thus, when animals are physically within an arm type, they can be seen as either naively exploring that arm or preferencing it due to the information previously gathered from exploration. Critically, when an animal is physically within an arm type, for example, the closed arms, they are also avoiding the open arms at the same time. Notably, avoidance behaviours such as freezing and stationary risk assessment also appear as flat regions of NEG curves of the time series of both arms, as well as the NEG curve for the total maze, effectively capturing this behaviour at a glance.

By measuring both avoidance and naïve exploration of an arm type, NEG captures the motivational conflict that governs the assessment of ALB during exposure to the EPM. By refining the measurement of the physical placement by integrating exploration, NEG leverages the original logic used to construct of the maze but more faithfully maps the behaviours to ALB states (Walf & Frye, 2007). Physical placement, as the primary behavioural construct, contrasts with non-locomotive, secondary measures such as grooming, rearing, and defecation. These secondary measures have been employed in multivariate multimodal analysis of behavioural profiles during EPM testing; however, the direction of effect is inconsistent and uncorrelated with arm type, whilst also being sensitive to non-testing confounds such as stress and feeding regimes (Casarrubea et al., 2015; Cruz et al., 1994; Hogg, 1996; Kalueff et al., 2016). Furthermore, studies have identified distinct grooming subtypes and that rearing occurs more frequently in the early stages of testing, implying that these are complex behaviours beyond the scope of the test design (Rojas-Carvajal & Brenes, 2020). For these reasons, and in service to the primary goal of this methodology to provide a unified measure of ALB, NEG focuses solely on tracking physical placement within the maze.

**Fig. 2:**
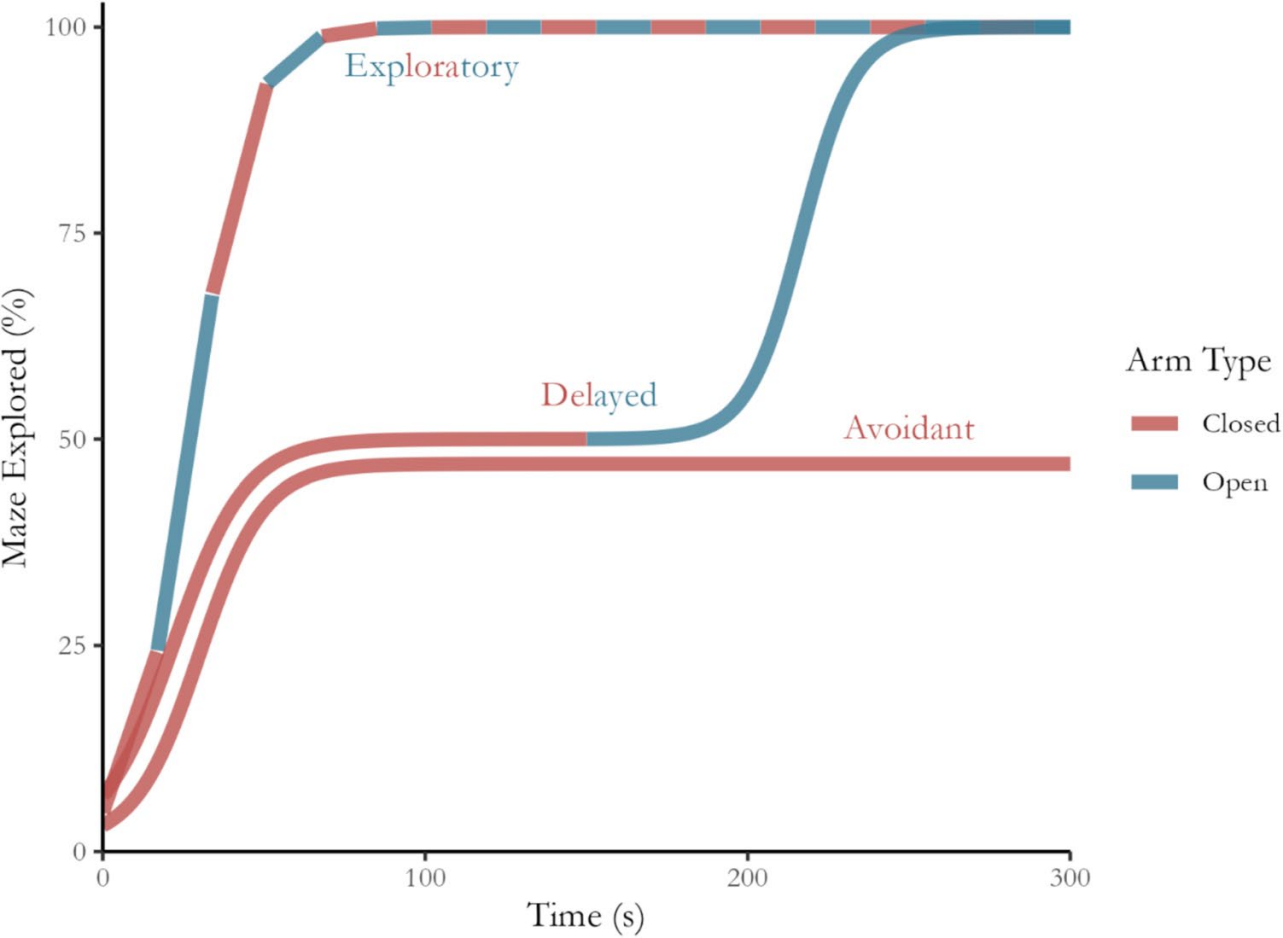
Exploratory, delayed and avoidant exploration phenotypes. The three behavioural phenotypes are distinguished by their differences over time and by the extent of open arm exploration. The line colour indicates which arm type is being explored during testing. *Note*: For illustrative purposes, the lines have been separated during the first 20 s across all phenotypes and up to 200 s for the delayed and avoidant phenotypes indicating higher overall ALB, but are equivalent during these periods.

NEG growth curves will differ between individual rodents as they vary in their level of anxiety-like behaviour. Nonetheless, these curves tend to match one of three distinct behavioural phenotypes: exploratory, delayed and avoidant. Given rodents’ innate preference for enclosed spaces, the delayed phenotype represents the typical behavioural pattern in untreated animals. This means that experimental interventions aimed at reducing anxiety-like behaviour typically manifest as reductions in the delay period or increases in subsequent open-arm exploration. Ideally, the most successful interventions should transition untreated rodents from their natural delayed phenotype to the exploratory phenotype as the timing and amount of exploration become indistinguishable between arm types. As shown in Fig. 2, a rodent exhibiting an exploratory behavioural phenotype traverses the open arms during the first phase of exploration, either exclusively or mixed with exploration of the closed arms. This pattern contrasts with an avoidant phenotype, where the rodent exclusively explores the closed arms, indicating a preference for safety. Critically, a rodent exhibiting the delayed phenotype treats the arms differently over time, initially avoiding the open arms and then transitioning into exploration of them. Although subsequent exploration of the open arms could occur in multiple phases, the key indicator of this phenotype is whether this exploration reliably occurs after a period of preferencing the closed arms. This preferencing causes a plateau in the curve, showing that exploration has ceased and that safer areas are preferred,

The growth curves in Fig. 2 highlight that ALB inference requires an estimate of both the phases of exploration and the proportion of the total area of the open arms that were explored. As outlined above, assessing the phases of exploration establishes whether a preference existed between arm types. For example, the closed arms being explored earlier than the open arms indicates greater ALB. In addition, measuring the depth of open-arm exploration is important because a low amount indicates a lack of physical commitment to exploring the area. This lack of commitment to physically exploring the open arms of the EPM indicates a high level of ALB, particularly when the depth is much lower than that of the closed arms.

**Fig. 3:**
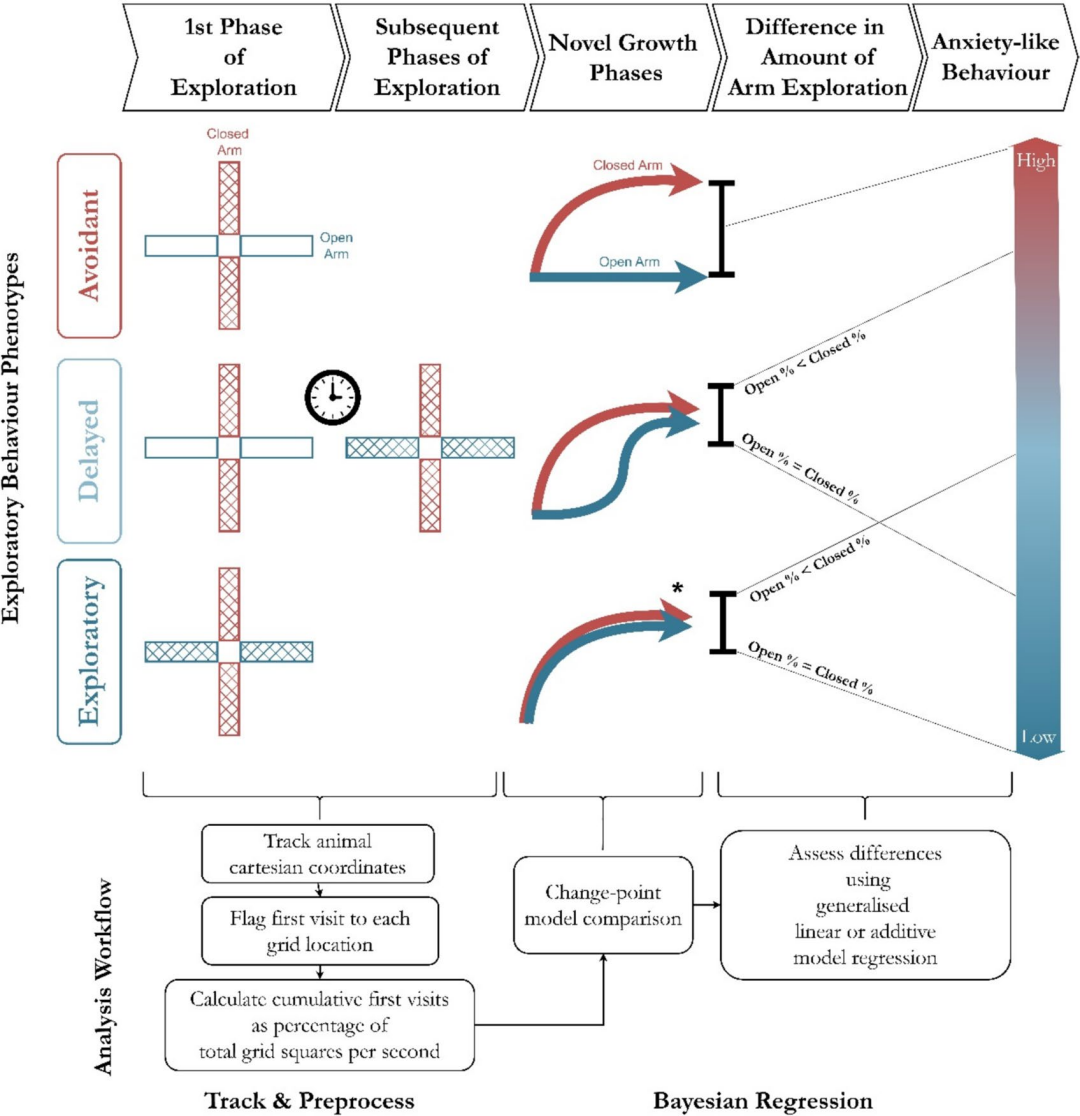
Mapping the exploratory behavioural phenotype to the ALB via novel exploration growth, including the analysis workflow. The position of a rodent in the maze is tracked and pre-processed to enable analysis of naïve exploration (left to right from the first phase of exploration to subsequent phases). To account for spatial differences between individuals or groups of interest, the cumulative first visits for the total maze and by arm type are evaluated via change-point models to establish their “novel growth phases”. The total amount of exploration was then compared between arm types to establish how much ALB was observed

Using the heuristics outlined above, Fig. 3 illustrates how the exploratory behavioural phenotype is mapped to a gradient of ALBs by examining the phases of exploration and the difference in the amount of exploration observed in each arm type. An avoidant phenotype is inferred when no open-arm exploration is observed and only the closed arms are explored, indicating the highest level of ALB. A Delayed phenotype displays a similar initial phase of closed-arm exploration, but open exploration only occurs in a separate subsequent phase. This sequence can be interpreted as indicating moderately-low to moderately-high ALB, depending on the extent to which the open arms were explored compared to the closed arms.

Conversely, an exploratory phenotype must explore a mix of arm types or open arms exclusively within the first exploration phase. This caveat exists because the critical element distinguishing this phenotype is the lack of initial avoidance of the open arms. The absence of avoidance indicates that exploration, rather than safety, motivates the traversal of this phenotype in this phase. Critically, the level of ALB inferred from this sequence still depends on the extent to which the open arms were explored compared to the closed arms. Low ALB is inferred where the amounts are similarly high; however, if the open arms are explored at a much lower comparative rate, then higher ALB is inferred.

Both conventional measures and NEG emphasise physical commitment to the open arms; however, NEG extends this emphasis by examining the depth and timing of commitment. When a rodent delays or abstains from exploring the open arms, this is considered to indicate that the safety of the closed arms provides more perceived benefit than the novelty of the unexplored open arms. Critically, a rodent demonstrates an exploratory phenotype when its first phase of exploration mixes arm types or when it explores only the open arms. This is because an exploratory rodent would treat the arms equally as novel areas and explore them agnostically. Conversely, a rodent displaying a greater ALB would show a preference for the closed arms, which would manifest as multiple exploratory phases where exploration of the open arms was delayed compared to that of the closed arms.

Casarrubea and colleagues (2013) claim that sequencing behaviours in time during testing in the EPM is critical to its construct validity. However, recent models ignore how behaviours are interrelated over time. (Arantes et al., 2013; Tejada et al., 2010). This oversight makes them incompatible with the dramatic changes that occur after information has been gathered and marginal value drops. This issue is further exacerbated by the lack of exploitative opportunities in the EPM, such as the presence of food, which renders the value of the open arms limited primarily to their initial novelty. Conversely, the inferential power of NEG in estimating the level of ALB is made possible through a simple decision framework that utilises both linear and nonlinear time series models.

## Method

The number of exploratory phases and NEG were estimated and compared using a multistage workflow (see “Analysis workflow” in Fig. 3). The workflow comprises two discrete sections: tracking and preprocessing and Bayesian regression analysis. Bayesian regression involves assessing the change points in the time series and analysing the conditional effects of interest, such as the effect of phenotype, arm, and phenotype-by-arm type interaction effects.

### Tracking and preprocessing

First, the position of the rodent’s centre of mass is tracked per second and translated into cartesian coordinates, with the centre of the maze being assigned as the origin (0,0). Various standalone programs, such as EthoVision and ANY-maze, can be used to track movement, as can packages such as pathviewr and TrackPy in R and Python. The remaining steps in the workflow were conducted in R using custom-developed scripts.

The coordinates of the rodent are then mapped to a unidimensional occupancy map using Cartesian coordinates with the origin at the centre of the maze. Each rectangle represents a 1 cm segment along the length of the arm and spans the entire width, capturing how far the animal has travelled into the arm (see Supplementary Figure S-1). Each segment is assigned a unique identifier to allow the timing of the animal’s first visit to that segment to be recorded. This process requires at least single-point tracking, typically of the animal’s centre of mass. However, multipoint tracking can also be used to measure exploration depth, particularly based on the position of the head, which is often extended near the edges of the arms. (Becker et al., 2023; Chetverikov & Verestoy, 1998). Critically, to accurately track the animal, the sampling rate of the tracking video must be high enough to map movement into each rectangle. A rate of 25 frames per second is recommended.

The cumulative percentage of the total maze or specific arm type explored is computed by aggregating the flagged grid rectangle per second. When expressed as a time series, this cumulative measure depicts the temporal phases in which this traversal occurs and the depth to which areas are explored during these phases via the percentage value. For ease of use, the custom scripts allow the user to manually indicate zone demarcations or use clustering across the dataset to automatically estimate the depth from the tracking value. Additionally, the centre zone is not included in any calculation as animals are placed there by researchers so no motivation can be inferred.

### Comparing phase models using change-point regression

**Fig. 4:**
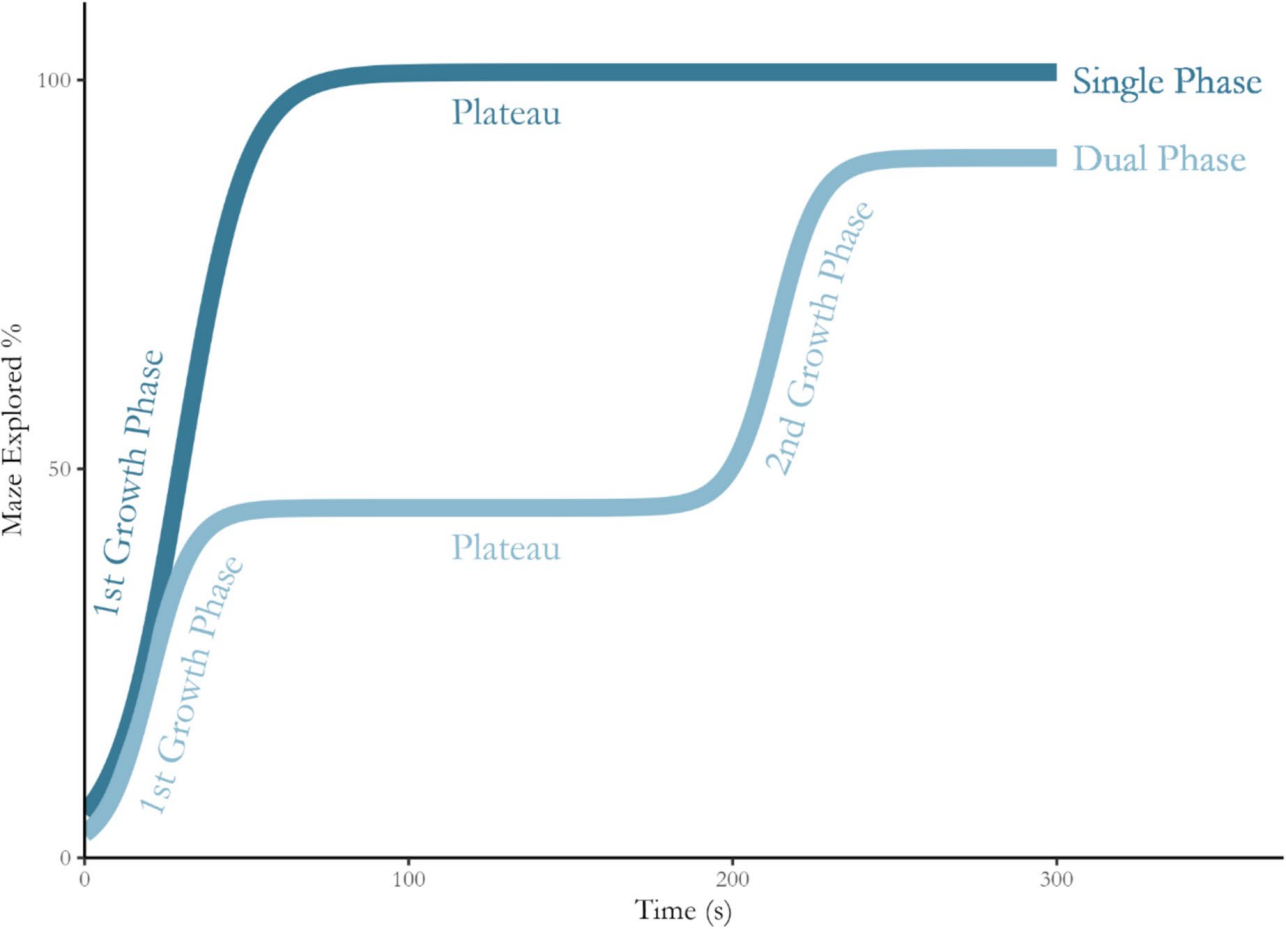
Single- and dual-phase exploration models for the maze. *Solid coloured lines* indicate novel exploration growth in the EPM over time for single- and dual-phase models. Both models show initial growth that plateaus with the dual-phase model characterised by a second growth phase

Bayesian change-point regression analysis is conducted on NEG over time for each phenotype’s total maze exploration and by arm type to evaluate the dynamics of the exploratory phase. Change-point models are fitted using the *mcp* package in R, which produces inferences regarding the location of changes in the trend of a time series (Lindeløv, 2020). Like other bounded exponential growth phenomena, this time series is modelled using an* n*-sized sigmoid function (Kucharavy & De Guio, 2015). As shown in Fig. 4, single and dual growth phase sigmoid functions are used to model change points in the NEG time series (see Supplementary S-2 for model specifications). In practice, the NEG time series for the entire maze and by arm type are modelled individually. The use of individual time series allows for inferences to be made about total maze and arm type-specific exploration.

### Estimating marginal and conditional effects using Bayesian regression

The current methodology follows the recommendations made by Betancourt (2020) when conducting Bayesian analysis for both change-point and generalised linear regression components of this workflow. Prior predictive checks are performed to assess the appropriateness of the chosen prior distributions. These checks involve generating data from the prior predictive distribution and evaluating whether these data are consistent with known or plausible values in the context of the study. Posterior predictive checks are performed to evaluate the model’s fit and its ability to reproduce observed data patterns. These checks involve visually comparing the observed data with data simulated from the posterior predictive distribution to assess model adequacy.

Model fit is also verified by ensuring that the parameter $\widehat{R}$ (Rhat) values are within the tolerance range of [0.9,1.1] and that the effective sample size (ESS) is greater than 1000 (Bürkner, 2017). Finally, leave-one-out cross-validation using Pareto-smoothed importance sampling (LOO_PS_-CV) is conducted using the loo R package to ensure adequate model fit (Vehtari et al., 2022). LOO_PS_-CV evaluates model fit by estimating the expected log posterior predictive distribution (elpd_loo_) and the effective number of parameters used to fit an out-of-sample dataset (Vehtari et al., 2017).

Change-point model comparisons are also made via LOO_PS_-CV. If both single- and dual-phase models meet the predictive checks and convergence criteria, models are ranked according to the highest elpd_loo_ with the lowest number of parameters. The model that returns the higher elpd_loo_ is preferred; when elpd_loo_ is equivalent, the most parsimonious model is selected.

Once phase model selection has been completed, the effects of interest are analysed using Bayesian mixed model regression. For the simulation below, the effects of interest included the marginal phenotype effect for the total maze and the conditional effect of arm type. Generalised linear mixed models can be used to estimate the effects of interest when growth curves of interest are best captured as single-phase growth curves. If any time series is best captured by a multiphase growth curve, such as the dual phase model specified above, then Bayesian GAM regression is applied to estimate effect characteristics. These models are fit via the *brms* package using smooth functions from the *mgcv* r package (Wood, 2011).

GAM regression is used because, as a semiparametric specification, it can model nonlinear relationships between the response and predictor variables very flexibly while still producing interpretable effect estimates via smoothing functions (Militão et al., 2023; White et al., 2020). The smoothing functions can be fit componentwise, for example, per phenotype or phenotype/arm type interaction, and are specified using a smoothing spline. For this workflow, a thin plate smoothing spline is chosen because it has excellent multidimensional properties, making it helpful in assessing spatiotemporal foraging patterns (Christensen-Dalsgaard et al., 2017; Preisler et al., 2004).

Bayesian mixed model regression analysis is conducted using the brms r package (Bürkner, 2018), with marginal and conditional effects being estimated using the *BayestestR* and *emmeans* packages or using custom functions (Lenth, 2022; Makowski, Ben-Shachar, & Lüdecke, 2019b). Marginal and conditional effects are evaluated inferentially using the Sequential Effect Existence and Significance Testing (SeXiT) framework, which reports estimates for centrality, uncertainty, existence, and significance (Kruschke, 2018; Makowski, Ben-Shachar, Chen et al., 2019a; Schwaferts & Augustin, 2020).

Centrality and uncertainty are reported using the median and highest density interval (HDI) of the distribution of the effect. The effect’s existence is assessed using the probability of its direction (PD). In contrast, significance is established by constructing a region of practical equivalence (ROPE), which verifies when an effect is negligible because it is practically equivalent to zero. To make the language synonymous with null hypothesis significance testing, the effect is reported as confirmed and equivalent to zero when greater than 97.5% of the posterior remains within the ROPE. Conversely, the equivalence of the effect to zero is rejected when less than 2.5% of the posterior remains within the ROPE. In cases where more than 2.5% but less than 97.5% of the posterior distribution of the effect remains within the ROPE region, the practical equivalence to zero is reported as undecided. For conventional measures, the ROPE region is constructed using the linear model specification $[ - 0.1 \times {SD}_{y}, 0.1 \times {SD}_{y} ]$. For growth models, the ROPE region is constructed using the logistic model specification $[- 0.18, 0.18]$. For further details on creating a region of practical equivalence, see Kruschke (2018) and Schwaferts and Augustin (2020).

### Simulating phenotypical exploratory and conventional behaviours

A simulation study was conducted to demonstrate how the change-point model comparison and regression analysis elements of the NEG workflow can be applied to samples of interest. Specifically, this study analysed the total maze and arm-specific NEG for the three behavioural phenotypes proposed above to show how to use NEG to infer the level of ALB being observed. The exploratory behaviour for the entire maze was simulated using the double sigmoid function with a sigmoid per arm type (see Supplementary Figure S-3 for parameter values for growth rates and inflexion points by phenotype for the total maze by arm type). Parameter values were chosen because they produce reasonable exemplars for the expected time series for each phenotype. The exploratory phenotype is expected to show rapid, arm-agnostic exploration growth at test onset; thus, arm growth rates are sampled from the same distribution, as are the inflexion time points for each arm. Conversely, the Delayed phenotype is expected to show an earlier inflexion time point for the closed arm than for the open arm. Finally, the growth rate and the inflexion points for the open arm exploration of the avoidant phenotype are 0.

**Fig. 5:**
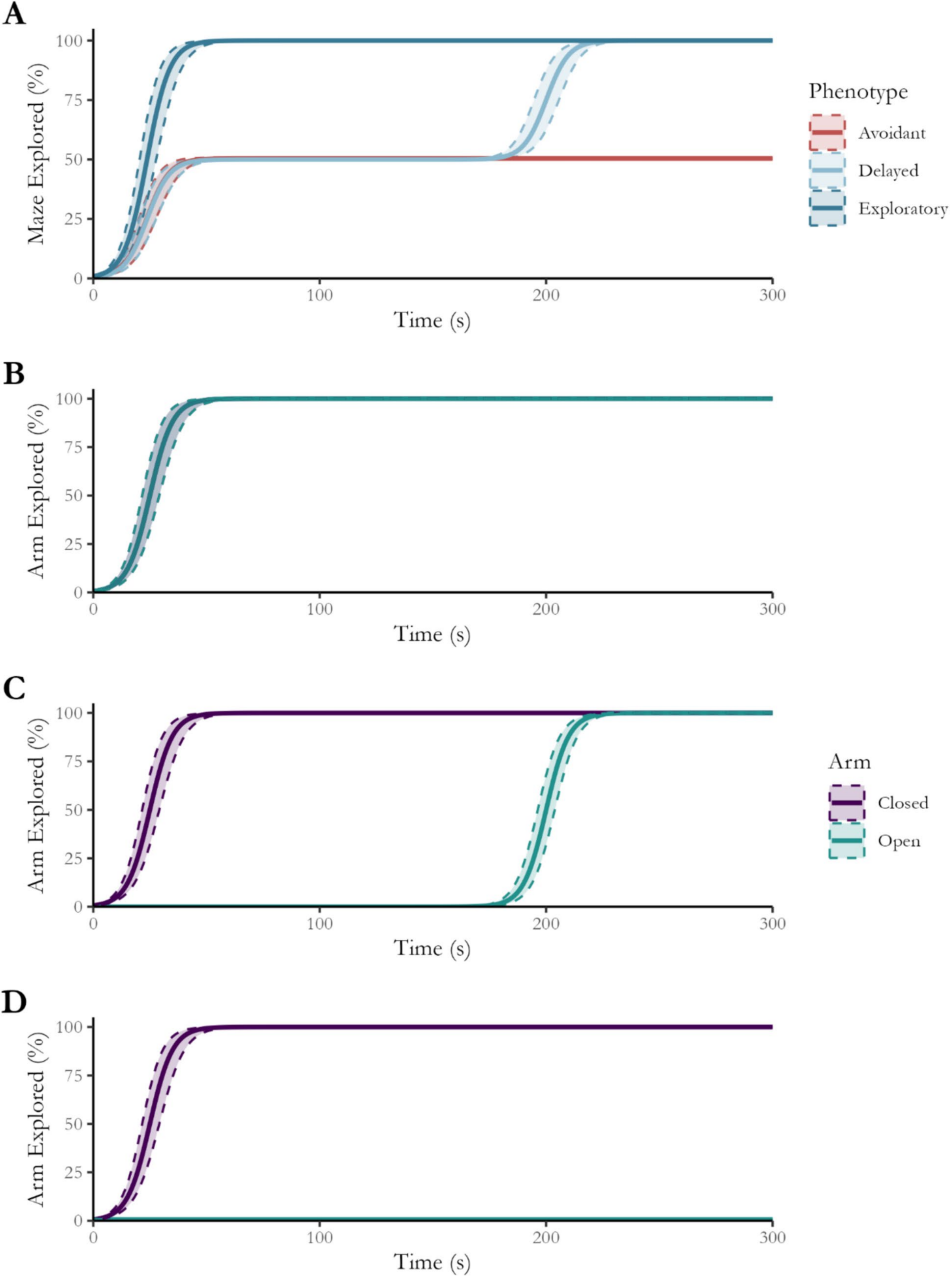
Total maze exploration and exploration by arm type per phenotype. All phenotypes initially showed similar rates of exploration growth for the entire maze (**A**); however, as further evidenced by the NEGs by arm type, the exploratory phenotype arm type growth curves overlap (**B**), while those for the delayed (**C**) and avoidant (**D**) phenotypes displayed differences over time due to their subsequent exploration or complete avoidance of the open arms

Figure 5 visually depicts the simulated NEG by phenotype for the total maze (A) and by arm type for all three phenotypes (B:D). As expected, the exploratory phenotype showed greater total exploration growth due to being arm-agnostic (B). In contrast, only closed arms were initially explored by the delayed (C) and avoidant phenotypes (D). After 200 s of testing, the delayed phenotype fully explored the open arms, reaching an equivalent total number of entries to the exploratory phenotype.

To demonstrate how NEG can address inconsistencies in conventional ALB measurements using the three phenotypes, we simulated their latency to enter, number of entries and total time spent in the open arms. To achieve this, we applied two key simulation heuristics:

- 1. The average velocity is assumed to be identical regardless of the arm type; Movement speed was kept identical between phenotypes since differences can signify illness, drug side effects, or malaise, which confound inferences (Schrader et al., 2018).
- 2. The first entry into the open arms is followed by a full exploration of that arm type, which is then followed by an immediate exit.

By ensuring that the first entry is followed by full exploration, we can accurately measure the latency to enter and the number of entries of all three phenotypes in their simplest forms. Critically, if we introduced additional features like shallow entries, this may make the simulation more realistic; however, the goal of this set of simulations is not complete realism but to use clear, qualitatively different phenotypes to show how conventional measures often confuse and contradict each other when estimating the ALB-associated behavioural patterns. Thus, given that the only difference between the exploratory and delayed phenotypes is a temporal delay in exploration, we only need to shift the timing of the first entry and keep all other variables unchanged.

Therefore, we only need to increase the latency to enter the open arms to highlight the contradiction between the equality of the entries and time spent in the open arms, and the latency to enter them. Specifically, if we interpret latency to enter as strongly indicative of ALB, we may label the delayed phenotype as more anxious, yet the equivalence in open-arm time and number of entries might suggest otherwise. By comparing this confusion to the coherency of NEG, we will show users can coherently infer ALB from their tracking data using the language of the phenotypes and the percentage of exploration that is observed.

Given these criteria, the minimum number of open arm entries required by the exploratory and delayed phenotypes is two, as there are two arms per type, with time spent in the open arms also being equivalent since once a rodent enters, it moves at the same speed and fully explores the arm and immediately leaves. This immediate exit reflects the expected optimal foraging behaviour (Kilpatrick et al., 2020). Conversely, the latency to enter the open arms is deliberately different between the exploratory phenotype and the delayed phenotypes and is set to the mid-point in testing. Critically, whilst the timing of the first entry is arbitrary, it is purely instructive as it is intended to demonstrate how NEG can detect meaningful differences in ALB despite the phenotypes being indistinguishable using the other two conventional measures. Consequently, the first entry into the open arms could occur at any time, rather it is the timing and depth of the exploration phases that matter inferentially. In this simulation, the delayed exploration of the open arms makes the delayed phenotype qualitatively different to not only the exploratory phenotype’s exploratory phase, but also to its own exploration of the closed arms. In contrast, the avoidant phenotype does not enter the open arms at all, so the latency to enter is set to the maximum testing duration (300 s), with zero entries into the open arms being observed and no time spent within them.

The distributions by phenotype for each open arm measurement value were simulated 1000 times using a truncated Gaussian distribution between 0 and 300 s since, in the case of the time spent in the open arms and the latency to enter them; the values must be positive real numbers that do not exceed 300 s as this is typically the total testing duration in seconds (see Supplementary Figure S-4 for simulation value summary and generation distribution).

## Results

### Conventional open-arm measures

**Fig. 6:**
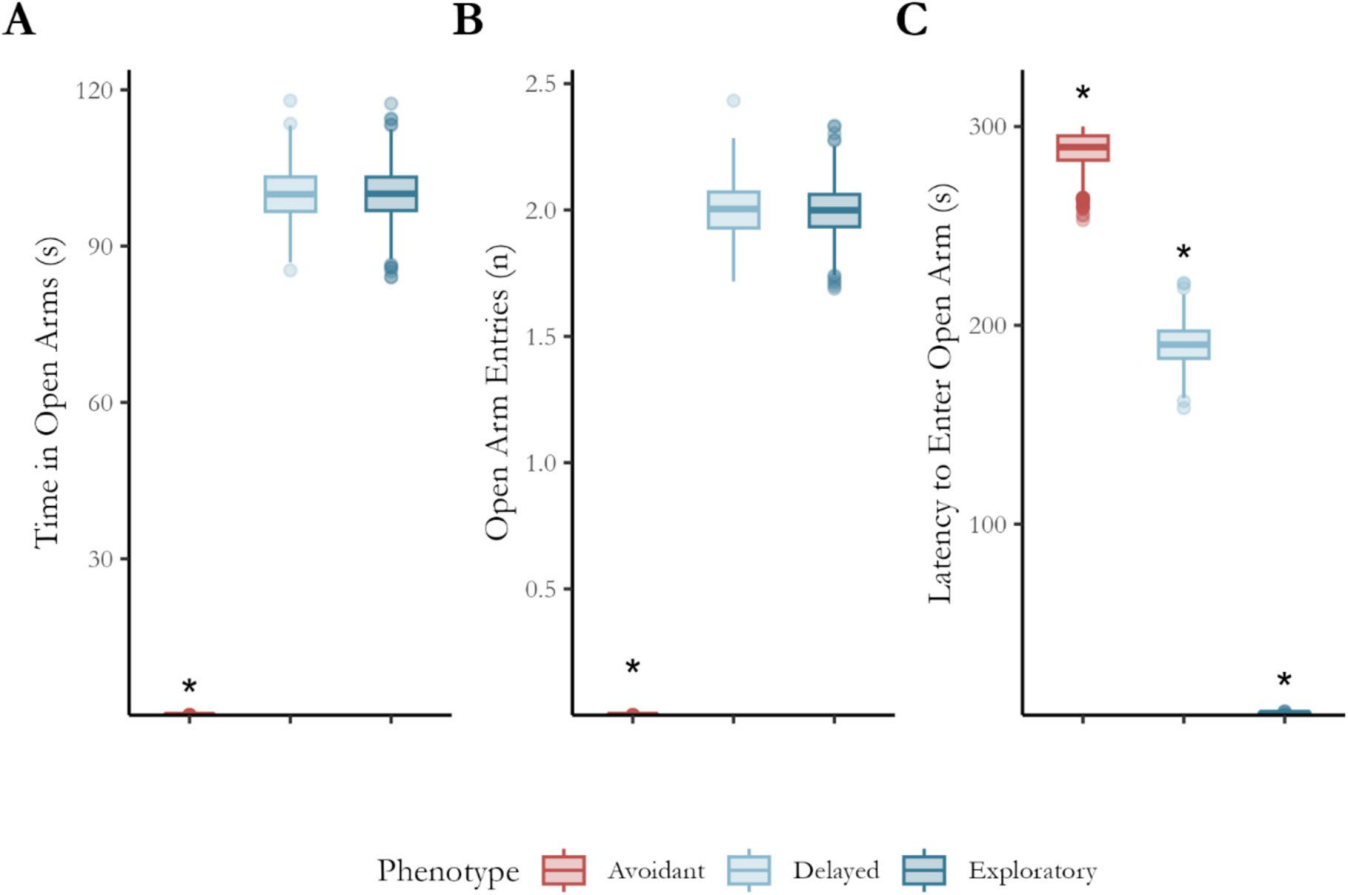
Mean and variance statistics for open arm preferencing metrics. The three phenotypes demonstrated differences in their latency to enter the open arms (**A**); however, only the avoidant phenotype displayed differences in the number of entries (**B**) into and time spent within (**C**) the open arms. An asterisk (*) indicates that it is not practically equivalent to any other phenotype

Bayesian linear regression was conducted using phenotype as a predictor to evaluate the effect of phenotype on conventional open-arm measures. Default priors were used for all parameters, and all models were well specified. Figure 6 illustrates the mean and variance of each conventional measure by phenotype, including the equivalence decision. As expected, the latency to enter the open arms of rodents with an avoidant phenotype, total entries and time spent within the open arms were not practically equivalent to the values measured using these parameters for exploratory or delayed phenotype rats. Conversely, only the latency to enter the open arms was not practically equivalent between the delayed and exploratory phenotypes (see Supplementary S-5 for effect summary and SeXit reporting table).

### Estimating novel exploration phases via change-point regression

To determine whether NEG occurred in a single or dual phase, two change-point regression models were fitted per phenotype to illustrate novel exploration across the entire maze (see Supplementary S-2 for full model specifications, including priors, and S-6:7 for prior predictive checks). The posterior distributions and model fit diagnostic criteria for all phenotypes can be found in section S-8 of supplementary materials. Using the diagnostic criteria outlined earlier, we determined that the single-phase model is the preferred choice for total maze NEG for the exploratory and avoidant phenotypes, whereas the delayed phenotype is best modelled using a dual-phase growth model.

### Comparing NEG between phenotypes for the total EPM

Given the heterogeneity of preferred phase models across phenotypes, mixed effects GAM regression was used to assess the effect of phenotype on NEG for the total maze (see Supplementary Figure S-9 for full model specifications, including priors and S-10 for posterior predictive checks and model diagnostics).

**Fig. 7:**
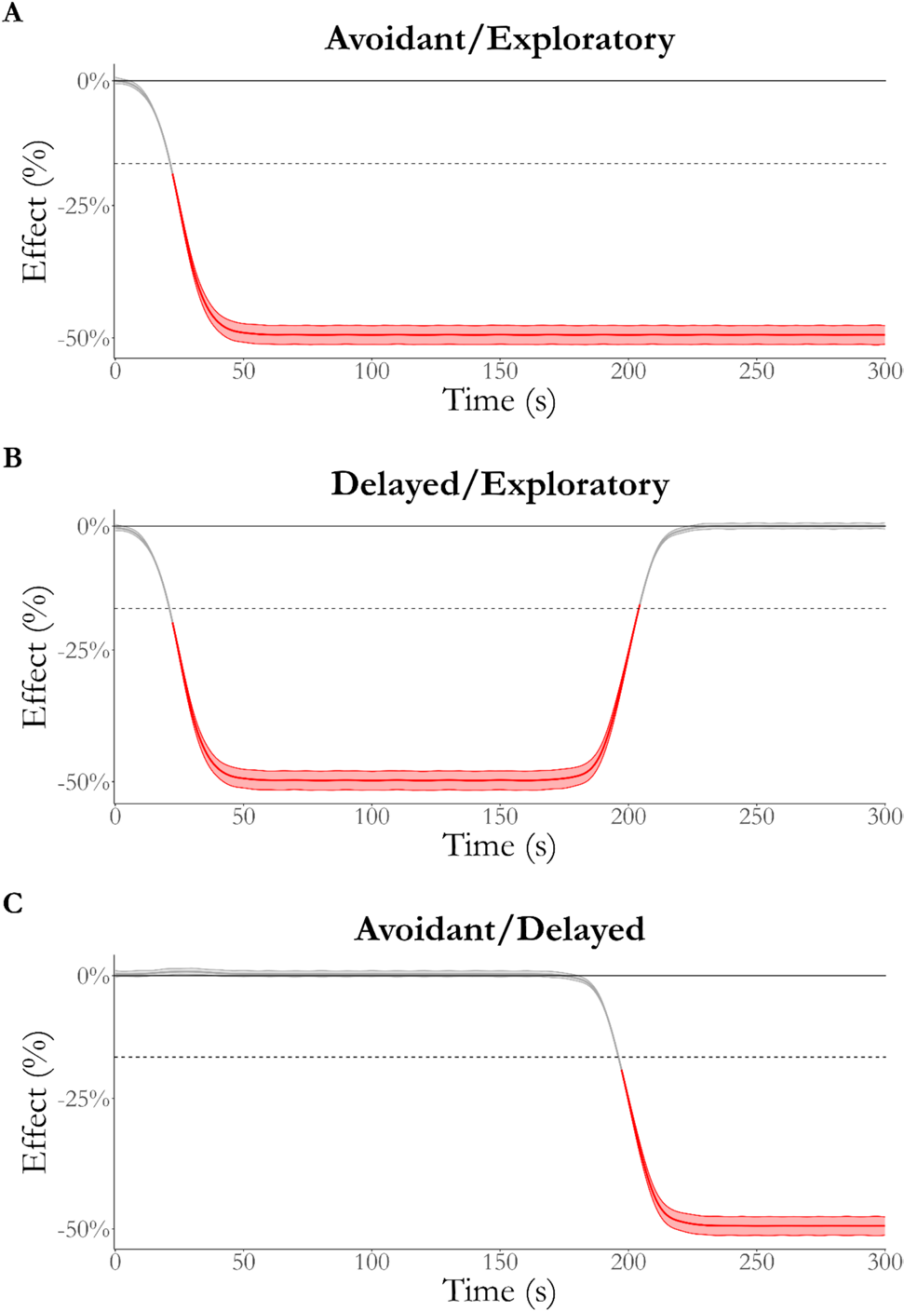
Effect of phenotype over time on total maze NEG. The avoidant and exploratory phenotypes (**A**) are not practically equivalent beyond the first few moments of the testing, whereas the difference between the exploratory and delayed phenotypes (**B**) is not equivalent for the first 200 s. The difference between the avoidant and delayed phenotypes is equivalent to zero for the first 200 s of testing (**C**). *Red* indicates that the effect is not practically equivalent to zero. *Note*: ROPE Region = 0 ± 18%, as indicated by the *dashed line*

Figure 7 shows the posterior of the marginal effect of phenotype over time for the total maze. The NEG for the exploratory phenotype is greater than that for the avoidant (A) and delayed (B) phenotypes, although this latter difference diminishes after 200 s. Conversely, the exploration growth of the delayed and avoidant phenotypes (C) is equivalent for the first 200 s of testing but diverges due to the phase of open-arm exploration by the delayed phenotype (see Supplementary S-11 for a full regression summary table).

### Comparing arm types between and within phenotypes

To determine whether NEG by arm type for each phenotype occurred in single or dual phases, the change-point regression used for the total maze exploration was applied to these contrast pairs (see Supplementary S-12 for posterior predictive checks and model diagnostics). As expected, all phenotype/arm exploration contrast pairs were best modelled as single-phase growth curves.

Given that all contrast pairs were best modelled using a single phase of exploration growth, a generalised linear mixed model was used with a binomial link function to assess conditional differences in hierarchical data such as subject-level time series (see Supplementary S-13 for a full model expression including priors and S-14 for a regression summary table including diagnostic values).

**Fig. 8:**
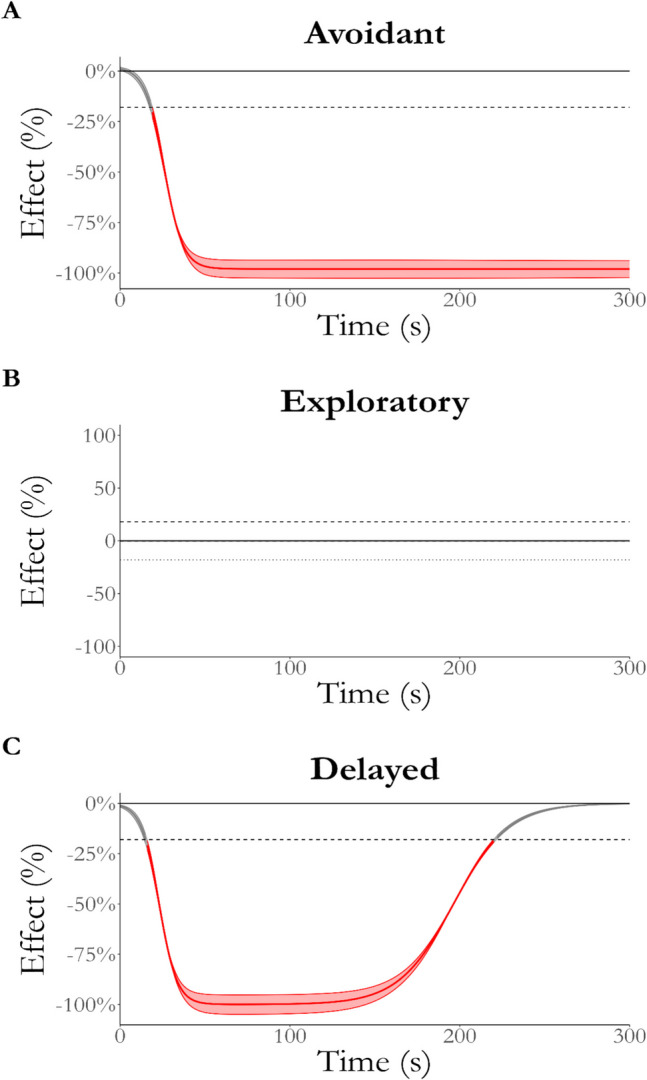
Effect of arm type for each phenotype over time. The difference between closed- and open-arm NEG for the avoidant phenotype (**A**) quickly reaches 100%, which contrasts with the effect for the exploratory phenotype (**B**), which is practically equivalent to zero for the duration of testing. Finally, the effect of arm type for the delayed phenotype initially reaches 100% but reduces back to practical equivalence after ~ 225 s. *Red* indicates that the effect is not equivalent to zero, where the *thick line* indicates the median effect over time and the *thinner lines* indicate the region of the full posterior of the effect. ROPE Region = 0 ± 18%, as indicated by the *dashed line*

Figure 8 shows the effect of arm type over time between each phenotype. As expected, the conditional effect of arm type rapidly became negative for the avoidant phenotype (A). Conversely, the effect of arm type is always equivalent to zero for the exploratory phenotype (B) and becomes equivalent to zero for the delayed phenotype in the later stages of testing (C).

**Fig. 9:**
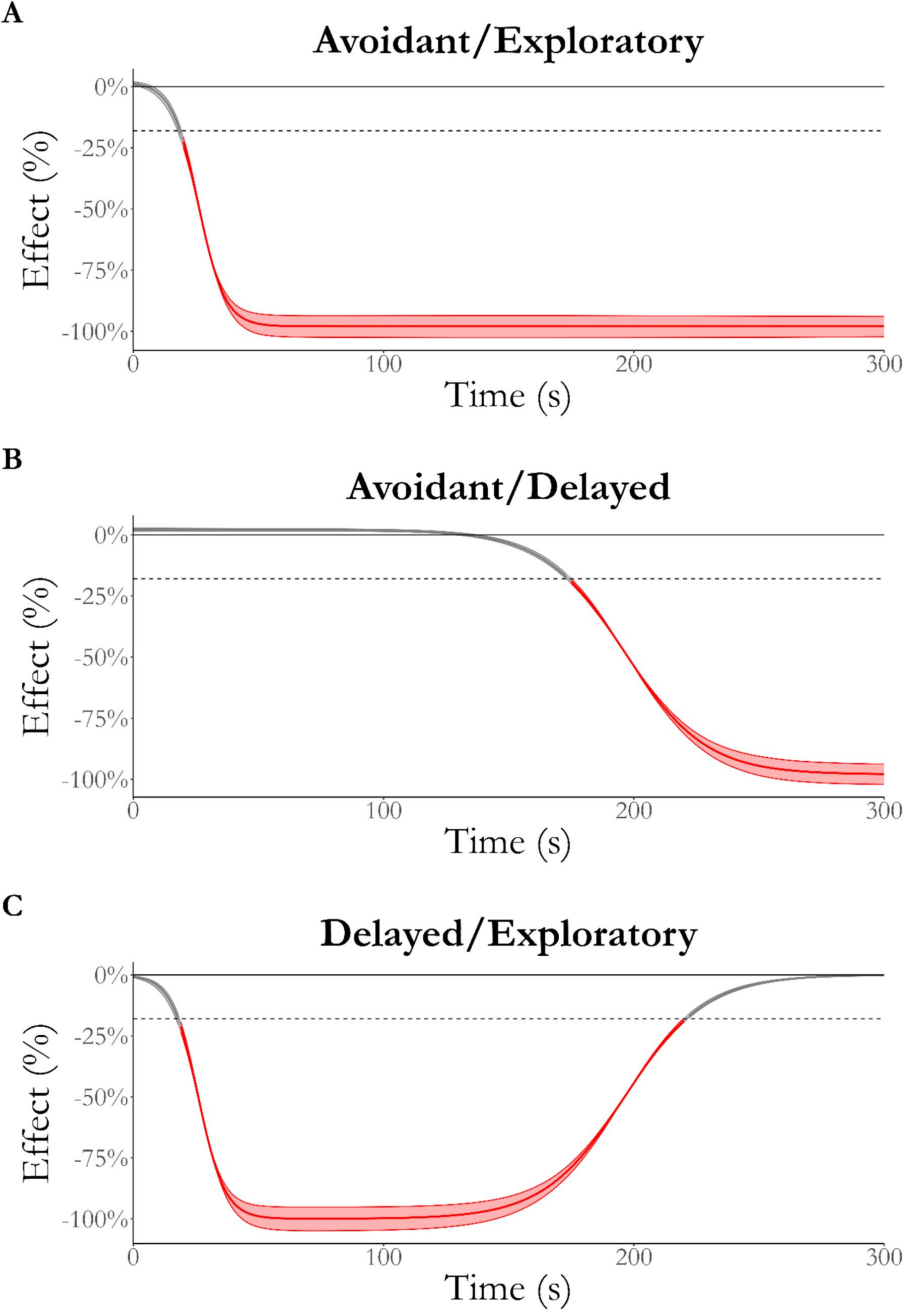
Effect of phenotype on open-arm NEG over time. The difference between the avoidant and exploratory phenotypes (**A**) quickly reaches 100%, while the effect reaches maximum later when compared to the delayed phenotype (**B**). Finally, the difference between the delayed and exploratory phenotype initially reaches 100% but reduces back to practical equivalence after ~ 225 s. *Red* indicates that the effect is not equivalent to zero, where the *thick line* indicates the median effect over time and the *thinner lines* indicate the region of the full posterior of the effect. ROPE Region = 0 ± 18%, as indicated by the *dashed line*

Figure 9 shows the effect of phenotype on open-arm NEG over time. As expected, the difference between the avoidant and exploratory phenotypes rapidly reached 100% of open-arm NEG (A). Conversely, the difference between delayed and avoidant (B) became practically significant only after 185 s of testing, matching the second phase of open arm-only exploration by the delayed phenotype. In contrast, the delayed and exploratory phenotypes are practically equivalent only after 200 s of testing after being significantly different for the majority of testing. This demonstrates a difference between the phases of open-arm exploration by the exploratory and delayed phenotypes.

## Discussion

This paper aimed to provide a single, coherent measure of exploratory and avoidant behaviours of rodents in the EPM. To achieve this, we propose evaluating novel exploration growth over time across the entire area of the maze and within each arm type. By simulating three distinct exploratory behavioural phenotypes, we showed that NEG uniquely and coherently maps movement throughout the maze to levels of ALB. These inferences were produced by analysing the phases of rat traversal using change-point analysis and comparing these phases using GAM regression.

According to the NEG analysis, the simulation of the exploratory phenotype exhibits the least anxious behaviour, as the change-point analysis indicated that exploration occurred in a single arm-agnostic fashion. The simulation of the delayed phenotype showed more anxious behavioural responses compared to the exploratory phenotype. This is due to the two arm-specific phases of exploration that signalled initial avoidance of the open arm, which receded over time. This reduction in exploratory activity reflects a dynamic updating of threat imminence over time, achieved through information gathering, which lowers avoidance and promotes exploration of the open arms (Davidson et al., 2019; Nonacs, 2001). The simulation of the avoidant phenotype displayed maximal ALB since it displays a continuous aversion to the open arm regions of the EPM. This continual aversion indicates that the naïve predicted value of the open arms never exceeds that predicted value of the safety provided by the closed arms.

Theoretically, a higher ALB is inferred when familiar, safer areas are preferred, while novel, riskier areas are avoided (Montgomery, 1951; Walf & Frye, 2007). In the EPM test, this behavioural pattern is partially observed by measuring the latency to enter the open arms, as it directly captures the avoidance of the riskier areas; however, it cannot substantiate the preferencing of safer ones. Despite this, the latency to enter the open arms was still able to correctly indicate that the delayed phenotype displayed higher ALB than the exploratory phenotype. Conversely, the NEG purposefully identifies when closed arms are not only being explored but are also preferenced prior to an initial entrance into the open arms. An example of preference is depicted by the delayed phenotype’s effect on NEG between arm types, where the time series showed that exploration growth plateaued before exploration of the open arms.

In contrast to the latency to enter the open arms, the conventional measure of the total number of entries into the open arms could not differentiate between the levels of ALB displayed by the delayed and exploratory phenotypes using their respective simulated values. Instead, analysis of this measure erroneously indicated that the two phenotypes were practically equivalent. Notably, this failure would become misleading if the delayed phenotype made even one additional, shallow entry into the open arms prior to exploring them. In this hypothetical case, all three conventional measures would lead to orthogonally different interpretations: latency to enter the open arms indicating that the delayed phenotype was more anxious, while the total time spent in them indicating no difference in anxiety-like behaviour and the total number of entries indicating lower anxiety-like behaviour. Critically, there is no justifiable way to select the optimal parameter from the three conventional measures because they evaluate different facets of ALB (Cruz et al., 1994; Walf & Frye, 2007).

The current study demonstrated that measuring physical exploration across time can isolate early avoidance from eventual exploration. This strength was evidenced by the successful identification of the dual phases of the simulated NEG across the entire maze by the delayed phenotype. Critically, when evaluated using NEG trends, an early, shallow entry into the open arms is correctly distinguished from the true, deep exploration displayed by the exploratory phenotype. This is because a shallow entry is reflected as no growth in the NEG curve, whereas a deep entry appears as strong growth. This contrast remedies inconsistencies that arise when using the three conventional measures.

A principal limitation of the current methodology is that the speed of movement can affect the calculation of NEG. When a rat is moving very quickly, typically at speeds greater than 25 cm/s, resolving the areas it has entered becomes difficult. At these speeds, novel exploration estimation requires higher resolution grids and frame rates for accurate tracking. Critically, this lack of resolution in the animal’s position is justified, as the faster the animal moves, the less information it can gather about any individual location. Given that the etiological basis for exploration is as an information-gathering behaviour, the speed of traversal through an area should vary proportionally with the amount of information gathered about the locale. Therefore, while NEG estimation does carry a bias toward slower exploration, this reflects greater certainty that a location has been explored compared to higher movement speeds. This assertion has been validated through testing, where speeds above 25 cm/s, captured at 24 frames per second, do not significantly impact overall trends in real-world datasets. Despite this, end users should be aware that such speeds may result in small variances between the expected NEG values and the actual calculated values. This limitation also underscores the need to capture animals at an adequate frame rate to minimise the impact.

The current framework does carry further limitations, however, particularly in the implementation of the regression analysis. For example, while GAMs are flexible enough to handle noisy real-world data, they require stronger assumptions about how these temporal patterns map to ALB. In particular, the choice of the smooth function and the number of basis functions impact the fit and interpretation of the output and its generalisability (Ezhov et al., 2018; Harmening & Neuner, 2016). In contrast, we explored the use of fully specified sigmoid functions, which can make interpretation easier and more generalisable, but it led to label switching during fitting, so they were deemed inappropriate (Ngah et al., 2016; Park et al., 2019).

The effectiveness of NEG has been tested against real-world data, which will be presented in a series of follow-up publications, where NEG was able to isolate differences in ALB due to treatment. Additionally, the methodology will be further validated by users who apply it to data representing previously established anxiolytic interventions, such as benzodiazepines and antidepressants (Hogg, 1996; Kurt et al., 2000).

A crucial limitation of NEG is that it only requires users to explicitly model the initial avoidance and exploration of an area or arm type. As noted previously, informed preferencing does occur when previously explored areas are revisited. Preferencing can be inferred indirectly by comparing differences in growth between arm types, as shown by the plateau between exploration phases demonstrated by the delayed phenotype. Despite this, further extensions could introduce more complex models that directly account for both exploratory and preferential states, such as using partially observable Markov decision processes to incorporate memory and agency into the model. Additionally, NEG emphasises physical displacement and presence as critical indicators of anxiety during EPM testing. Future studies may incorporate dynamic Bayesian networks that include non-locomotor behaviours such as grooming or rearing to increase the resolution of the multidimensional presentation of anxiety during exposure to the EPM. Finally, the technical aspects of the workflow may pose a barrier to implementation for some EPM users. To enhance ease of use, the code used in this workflow is publicly available at https://github.com/MZelko82/NEG. This repository contains extensive commentary that includes suggestions for substituting frequentist alternatives when the user prefers them, as well as discussions of theoretical and computational differences between the methods. It also contains an interactive simulation of possible time series for each of the three phenotypes, allowing the user to more easily map their behaviour in the maze to the NEG time series curves.

Additionally, users familiar with linear regression should find the shift to GAMs reasonably smooth, as the latter allows the linear parameters to accommodate flexible, non-linear functions in their place (Zuur et al., 2007). As a result, the computation of marginal and conditional effects closely parallels that of linear models, and the accompanying code guides users through running and interpreting the necessary functions.

## Conclusion

The present study demonstrated that novel exploration of the EPM accurately distinguished between three phenotypical anxiety-like behaviours. This capability was achieved using Bayesian change-point and GAM regression to model rodent behavioural data from EPM assays. The study also demonstrated that conventional ALB measures produce inconsistent and erroneous inferences when analysed using simulated data representing phenotypical exploratory behavioural patterns.In contrast, NEG produces a unified and coherent measure of ALB when assessing rodents in the EPM.

## Supplementary Information

Below is the link to the electronic supplementary material.

Supplementary file1 (PDF 2206 KB)

## Data Availability

Not applicable.

## Code availability

The code used to calculate NEG and replicate the analysis workflow is available at https://github.com/MZelko82/NEG.
